# PD-1/PD-L1 expression profiles within intrahepatic cholangiocarcinoma predict clinical outcome

**DOI:** 10.1186/s12957-020-02082-5

**Published:** 2020-11-23

**Authors:** Lingyu Tian, Jiaqiang Ma, Lijie Ma, Bohao Zheng, Longzi Liu, Danjun Song, Yining Wang, Zhao Zhang, Qiang Gao, Kang Song, Xiaoying Wang

**Affiliations:** 1 Department of Liver Surgery and Transplantation, Liver Cancer Institute, Zhongshan Hospital and Key Laboratory of Carcinogenesis and Cancer Invasion, Fudan University, 180 Fenglin Road, Shanghai, 200032 China; 2 Department of General Surgery, Zhongshan Hospital (South), Shanghai Public Health Clinical Center, Fudan University, Shanghai, 200083 China; 3 Department of General Surgery, Zhongshan Hospital, Fudan University, 180 Fenglin Road, Shanghai, 200032 China; 4 Department of General Surgery, The First Affiliated Hospital of Nanchang University, Nanchang, 330000 Jiangxi China; 5 Department of Hepatobiliary Surgery, The First Affiliated Hospital of Fujian Medical University, Fuzhou, 350001 Fujian China

**Keywords:** Intrahepatic cholangiocarcinoma, Programmed cell death protein-1, Programmed cell death protein ligand 1, Immunotherapy, Tumor immune micro-environment

## Abstract

**Objective:**

Immunotherapy targeting the programmed cell death protein-1 (PD-1)/programmed cell death protein ligand 1 (PD-L1) pathway has been observed to be efficient in several solid tumors. We aim to investigate the prognostic significance of PD-1/PD-L1 expression profile in intrahepatic cholangiocarcinoma (ICC).

**Materials and methods:**

We investigated the expression of PD-1, PD-L1, CD8^+^ T cells, and CD68^+^ macrophages in paired tumor and adjacent normal tissues from 322 ICC patients using tyramide signal amplification (TSA)-based multiplexed immunohistochemistry.

**Results:**

We found that high proportion of tumor-infiltrating CD8^+^ PD-1^High^ within CD8^+^ PD-1^+^ T cells significantly correlated with advanced TNM stage (*P* = 0.035). ICC patients with high proportion of CD8^+^ PD-1^High^ in CD8^+^ PD-1^+^ had worse postoperative survival than low proportion patients (*P* = 0.0037), which was an independently prognostic factor for OS (*P* = 0.025,). The density of CD68^+^ PD-L1^+^ significantly and positively correlated with the density of CD8^+^ PD-1^High^ (*P* < 0.0001, *r* = 0.5927). The proportion of CD68^+^ PD-L1^+^ within CD68^+^ ICC was the risk factor for OS and TTR but not an independently factor for prognosis. The CD68^+^ PD-L1^+^ macrophages and CD8^+^ PD-1^High^ T cells may cooperatively play a role in inhibiting anti-tumor immunity.

**Conclusion:**

CD68^+^ PD-L1^+^ macrophages and CD8^+^ PD-1^High^ T cells predict unfavorable prognosis, which could also bring new progress about immune target therapy in ICC research.

**Supplementary Information:**

The online version contains supplementary material available at 10.1186/s12957-020-02082-5.

## Synopsis

This research addressed the prognostic value of PD-1/PD-L1 expression on CD8^+^ T cells and CD68^+^ macrophages in intrahepatic cholangiocarcinoma. The CD68^+^ PD-L1^+^ macrophages and CD8^+^ PD-1^High^ T cells may cooperatively play a role in inhibiting anti-tumor immunity. Immune target therapy may bring better oncological outcomes for ICC patients.

## Introduction

Intrahepatic cholangiocarcinoma (ICC), a highly aggressive biliary epithelial malignancy, roughly accounts for 10–15% of primary liver cancer, with an increasing disease morbidity and mortality worldwide [1, 2]. Risk factors of ICC mainly include cholangitis, biliary cirrhosis, hepatolithiasis, and viral hepatitis B/C, indicating that chronic inflammation may facilitate ICC development [3, 4]. Currently, hepatectomy remains the only potentially curative treatment for ICC patients, but the clinical outcomes of ICC are dismal, with the 5-year postoperative overall survival (OS) rate ranging from 20 to 40% [5, 6]. For those unresectable tumors, the current treatment effects on tumor control in patients remain limited. Therefore, effective therapeutic strategies are urgently needed.

As an inflammatory response, immune response can recognize and kill tumor cells in the process of immune surveillance [7]. Tumor cells naturally or adaptively express immunosuppressive molecules to evade the immune attack, which makes immune escape and immunosuppression play a vital role in the progression of tumor [8, 9]. Recently, immunotherapy that normalizes the immune response in the tumor microenvironment (TME), especially through targeting programmed cell death (PD) pathways, has been shown to achieve high response rates in several malignant tumors [10]. The TME is a complex multicellular functional compartment that includes tumor cells, stromal cells, and immune cells [11]. Macrophages and CD8^+^ T lymphocytes, as part of immune cells in tumor microenvironment, are common in intrahepatic cholangiocarcinoma, and significantly affect the occurrence of intrahepatic cholangiocarcinoma [12]. A previous study has demonstrated that tumor infiltrating lymphocytes (TILs) are associated with prognosis in patients with ICC, and CD8^+^ infiltration is associated with better survival and lower recurrence [13]. Furthermore, CD68^+^ macrophages can affect the progression of tumors with conflicting results. Some studies have reported that high levels of tumor-associated macrophages (TAMs) are associated with better survival in patients with ICC [14], while others showed opposite trends [15].

Immune checkpoints maintain self-tolerance and protection of normal tissues during the immune response, but these checkpoints are often altered by cancer cells to avoid epidemic surveillance [16]. The programmed cell death protein-1 (PD-1)/programmed cell death protein ligand 1 (PD-L1) interactions between tumor cells and CD8^+^ T cells were discovered in hepatocellular carcinoma (HCC), where it was found that an increased distribution of CD8^+^ PD-1^+^ T cells in tumors predicted poor disease progression and postoperative recurrence [17]. Furthermore, the expression of PD-1 in TAMs inhibits phagocytosis and tumor immunity has been reported [18].

There are only a few data available concerning ICC immunotherapy [12]. One previous study has revealed that PD-1/PD-L1 was overexpressed in ICC, and the expression of PD-L1 in the frontier of tumors was associated with a 60% reduction in survival [19]. One case report shows that two recent ICC patients achieved complete remission after PD-1 blockade [20]. In the tumor microenvironment of ICC, there is limited research on the clinical impact of immune cells such as macrophages, lymphocytes, and immune checkpoints. In this study, we investigated CD68^+^ in macrophages, CD8^+^ T cells, and PD-1/PD-L1, as well as immune cell subgroups classified on PD-1/PD-L1 expression, for their association with clinical features and prognosis of ICC patients, providing some meaningful information for ICC oncological research.

## Materials and methods

### Patient selection

A total of 322 paraffin-embedded ICC tumor and matched non-tumor tissues were collected from the Liver Cancer Institute, Zhongshan Hospital, Fudan University (Shanghai, China), from 2005 to 2011 [21]. The processing of all sample information meets the requirements of the Declaration of Helsinki. No distant metastases were found in this series, and no prior anti-tumor treatments were performed before surgery. Clinicopathologic variables include age, gender, HBsAg history, liver cirrhosis, carbohydrate antigen 19-9 (CA19-9), Child-Pugh classification, tumor number, size, lymph node metastasis, tumor TNM stage, and microscopic vascular invasion (MVI), which were detailed in Supplementary Table 1. All patients provided signed informed consent.

### Follow-up strategy

Follow-up strategy after surgery was along the lines of our previous standard [22]. Overall survival (OS) was the duration from the day of surgery to death of the patients. Nevertheless, if patients were still living by the time of our last follow-up, data of ICC patients were censored [21]. Time to recurrence (TTR) was described as the duration from the date of surgery to confirm the recurrence of the tumor in the relapsed patients, or from the date of surgery to the date of the last observation of the non-relapsed patients [23]. The deadline of the follow-up data collection is October 31, 2016.

### Tissue microarray (TMA) construction and multiplex immunohistochemistry

Duplicated tissue cores of 1-mm diameter were obtained from a different area of the same tissue block in tumor and peri-tumor based on H&E staining (designated as intra-tumor and peri-tumor, respectively, a total of four cores). TMA were constructed as previously described [24]. Multispectral immunohistochemistry was performed as previously described in detail in the PerkinElmer Opal kit^R^ protocol. The antigenic binding sites were visualized using Opal dyes: Opal-570, Opal-540, Opal-650, and Opal-620 were applied to each antibody (Supplementary Figure 1A).

### Tissue imaging and image analysis

The PerkinElmer Vectra platform (Vectra 2.07 and 3.03; PerkinElmer) was utilized to exam multiplexed immunostaining TMA slides [25]. The inForm Advanced Image Analysis Software (inForm 2.2.1; PerkinElmer) was implemented to unmixing each reagent from the multispectral photographs by utilizing the spectral archives formed from single-colored photographs [25]. R script was applied for quantification of positively stained cell. The gating or statistical analysis strategies were described as previously [17].

### Statistical analysis

All statistical calculations were implemented by using SPSS statistic software (v20, IBM, Armonk, NY) and Prsim Graphpad 8.0 (GraphPad Soft Inc., San Diego, CA). Paired *t* test, McNemar chi-square test, and Fisher’s exact test were applied to determine statistically significant differences between paired data. On the basis of outcome (death and recurrence), we utilized ROC curves and Youden index to delimit the optimal cut-off values. OS and TTR was estimated by Kaplan-Meier curves, and differences between groups were compared by log-rank tests. The Cox regression model was applied to evaluate the univariate and multivariate analyses. Variables with a *P* value < 0.05 according to univariate analyses and those considered to be clinically relevant were included in the multivariate analyses. A two-tailed *P* < 0.05 was regarded as statistically significant.

## Results

### Baseline characteristics of ICC patients

The clinicopathological characteristics of 322 ICC patients were described in Supplementary Table 1. The median OS time was 27.2 months (range 2.6–60.0 months), and the median TTR time was 12.8 months (range 1.0–60.0 months). The overall cumulative survival rates at 1, 3, 5 years after surgery were 75% (95% CI 71.08–78.92), 45% (95% CI 39.12–50.88), and 34% (95% CI 28.12–39.88%), respectively. The cumulative disease-free survival rates at 1, 3, and 5 years after operation were 55% (95% CI 49.12–60.88%), 38% (95% CI 32.12–43.88%), and 35% (95% CI 29.12–40.88%), respectively. The follow-up data collection was completed on October 31, 2016. The median follow-up time was 27.0 months (range 1.0–60.0 months).

### Expression pattern and prognostic value of PD-1 and PD-L1 in ICC

**Fig. 1:**
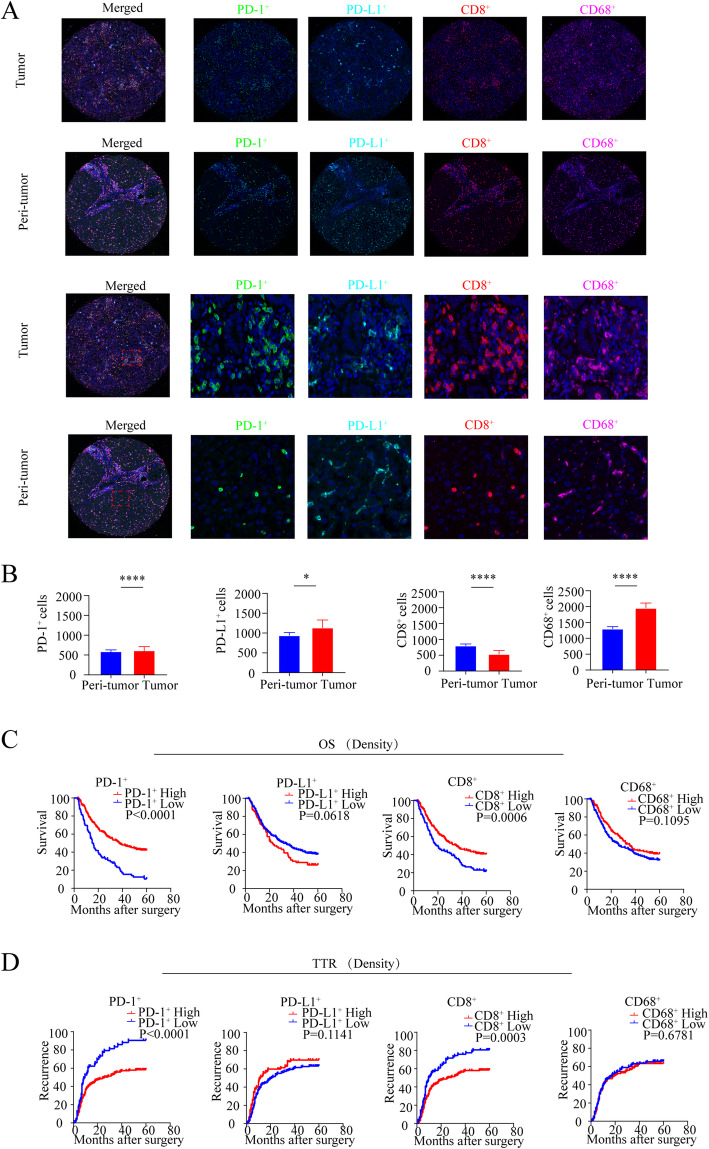
Distribution and prognostic significance of PD-1, PD-L1, CD8, and CD68 in tumor and peri-tumor by multiplex immunohistochemistry in ICC patients. **a** Representative multiplex immunofluorescence staining with the indicated immune markers in ICC tumor and peri-tumor tissues: green (PD-1), cyan (PD-L1), red (CD8), purple (CD68), and blue (DAPI), respectively. Scale bar, 50 μm. **b** Quantitative comparisons analysis of the density of PD1^+^, PD-L1^+^, CD8^+^, and CD68^+^, between paired tumor tissues (T) and peri-tumor tissues (P) (Wilcoxon signed-rank test). **c**, **d** Kaplan-Meier analysis of overall survival (OS) (**c**) and relapse-free survival (RFS) (**d**) in ICC according to the density of PD1^+^, PD-L1^+^,CD8^+^, and CD68^+^ in the 322 ICC patients

To fully investigate the expression pattern, staining of PD-1/PD-L1 in ICC tissues was performed using TSA-IHC and analyzed using the inForm system (Fig. 1a). Quantitatively, the comparison of PD-1 expression between the whole tumor and non-tumor cores showed significantly higher expression in tumor tissues than in peri-tumor tissues (*P* < 0.0001). Similarly, PD-L1 expression was higher in tumor tissues than in peri-tumor tissues (*P* = 0.0123) (Fig. 1b). The PD-1 was solely expressed on the non-tumor cells; however, the PD-L1 was primarily expressed on both tumors and non-tumor cells.

To evaluate the clinical importance of PD-1/PD-L1, we estimated the Youden index of PD-1/PD-L1 expression and classified the ICC patients into high and low expression groups on the basis of a cut-off value of positive immunostaining in tumor tissue. As detailed in Table 1, high expression of PD-1 in tumor correlated with low CA19-9 level (*P* = 0.006) and smaller tumor size (*P* = 0.015). High expression of PD-L1 in tumor correlated with the presence of lymph node metastasis (*P* = 0.006) and advanced TNM stage (*P* = 0.025).

**Table 1:** Correlation between the expression of PD-1, PD-L1, CD8, and CD68 and clinicopathological features

Characteristics	PD-1^+^			PD-L1^+^			CD8^+^			CD68^+^		
	Low	High	*P*	Low	High	*P*	Low	High	*P*	Low	High	*P*
Age, years												
< 60	41	132	0.965	124	49	0.120	54	119	0.646	100	73	0.551
≥ 60	35	114		118	31		43	106		91	58	
Gender												
Female	36	92	0.121	110	18	**0.000**	49	79	**0.010**	87	41	**0.010**
Male	40	154		132	62		48	146		104	90	
HBsAg												
Negative	55	144	**0.030**	153	46	0.361	67	132	0.078	125	74	0.104
Positive	21	102		89	34		30	93		66	57	
Cirrhosis												
No	58	178	0.495	181	55	0.290	72	164	0.803	142	94	0.606
Yes	18	68		61	25		25	61		49	37	
CA19-9												
Low (≤ 37)	28	135	**0.006**	128	35	0.156	39	124	**0.014**	96	67	0.876
High (> 37)	48	111		114	45		58	101		95	64	
Child-Pugh												
A	75	236	0.250	236	75	0.107	97	214	**0.027**	185	126	0.743
B	1	10		6	5		0	11		6	5	
Tumor size (cm)												
≤ 5	25	120	**0.015**	107	38	0.609	33	112	**0.009**	73	72	**0.003**
> 5	51	126		135	42		64	113		118	59	
Tumor number												
Single	57	187	0.857	188	56	0.164	72	172	0.670	154	90	**0.014**
Multiple	19	59		54	24		25	53		37	41	
LN invasion												
No	61	205	0.537	208	58	**0.006**	81	185	0.781	157	109	0.815
Yes	15	41		34	22		16	40		34	22	
TNM stage												
I	55	192	0.306	193	54	**0.025**	74	173	0.907	144	103	0.500
II–III	21	54		49	26		23	52		47	28	
MVI												
No	63	213	0.422	208	68	0.833	83	193	0.960	164	112	0.926
Yes	13	33		34	12		14	32		27	19	

Chi-square test and Fisher’s exact test were performed

*HBsAg* hepatitis B surface antigen, *CA19-9* carbohydrate antigen 19-9, *LN* lymph node, *TNM* tumor-nodes-metastasis, *MVI* microvascular invasion

We found that ICC patients with high expression of PD-1 has better OS (logrank test: *P* < 0.0001) and prolonged TTR (logrank test: *P* < 0.0001). However, high expression of PD-L1 neither correlated with ICC patients’ OS (logrank test: *P* = 0.0618) nor TTR (logrank test: *P* = 0.1141) (Fig. 1c, d).

In the univariate analyses, CA19-9(*P* = 0.001), tumor size (*P* = 0.003), number (*P* = 0.002), lymph node metastasis (*P* < 0.001), TNM stage (*P* < 0.001), and PD-1 (*P* < 0.001) were found to correlate with OS. In the multivariate analyses, PD-1 (*P* < 0.001, HR = 0.457, 95% CI 0.337–0.619) continued to be the prognostic factor of OS (Table 2).

**Table 2:** Univariate and multivariate analyses of prognostic features and overall survival

	OS			
**Variable**	**Univariate analysis**		**Multivariate analyses**	
	HR (95%CI)	*P*	HR (95%CI)	*P*
**Age (years)**				
<60 vs. ≥ 60	1.014 (0.770–1.335)	0.920		
**Gender**				
Male vs. female	1.144 (0.863–1.519)	0.350		
**HBsAg**				
Negative vs. positive	0.786 (0.590–1.046)	0.099		
**Cirrhosis**				
No vs. yes	1.195 (0.883–1.617)	0.249		
**CA19-9**				
Low (≤ 37) vs. high (> 37)	1.564 (1.188–2.059)	**0.001**		
**Child-Pugh**				
A vs.B	0.879 (0.390–1.981)	0.756		
**Size (cm)**				
≤ 5 vs. > 5	1.521 (1.149–2.013)	**0.003**		
**Number**				
Single vs. multiple	1.626 (1.196–2.209)	**0.002**		
**LN invasion**				
No vs. yes	2.752 (1.984–3.818)	**0.000**		
**TNM stage**				
I vs. II–III	2.310 (1.711–3.120)	**0.000**		
**MVI**				
No vs. yes	1.196 (0.816–1.752)	0.360		
**PD-1**				
Low vs. high	0.431 (0.320–0.580)	**0.000**	0.457 (0.337–0.619)	**0.000**^**a**^
**PD-L1**				
Low vs. high	1.332 (0.982–1.806)	0.650	NA	NA
**CD8**				
Low vs. high	0.615 (0.462–0.819)	**0.001**	0.629 (0.470–0.842)	**0.002**^**b**^
**CD68**				
Low vs. high	0.795 (0.599–1.055)	0.112	NA	NA
**CD8PD1**^**High**^**/CD8PD1**				
Low vs. high	1.736 (1.184–2.547)	**0.005**	1.557 (1.057–2.292)	**0.025**^**c**^
**CD8PD1**^**Low**^**/CD8PD1**				
Low vs. high	0.576 (0.393–0.845)	**0.005**	0.642 (0.436–0.946)	**0.025**^**d**^
**CD68PDL1/CD68**				
Low vs. high	1.388 (1.022–1.884)	**0.036**	1.111 (0.809–1.527)	0.514^e^

Cox regression model was performed

*HBsAg* hepatitis B surface antigen, *CA19-9* carbohydrate antigen 19-9, *LN* lymph node, *TNM* tumor-nodes-metastasis, *MVI* microvascular invasion

*a*, *b*, *c*, *d*, and *e CA19-9*, *size*, *number*, *LN* invasion, and *TNM stage* were adjusted in multivariate analysis

In terms of TTR, in the univariate analyses, tumor size (*P* = 0.006), number (*P* < 0.001), lymph node metastasis (*P* < 0.001), TNM stage (*P* < 0.001), MVI (*P* = 0.026), and PD-1 (*P* < 0.001) were found to correlate to TTR. In the multivariate analyses, PD-1 (*P* < 0.001, HR = 0.554, 95% CI 0.402–0.763) continued to be the prognostic factor of TTR (Supplementary Table 2).

Collectively, our data showed that PD-1 expression was an independent prognostic factor for OS and TTR, but PD-L1 failed to be a valuable prognostic factor for OS and TTR.

### Infiltrating and prognosis value of T cells, macrophages in human ICC

Next, we evaluated the clinical significance of T cells and macrophages. The density of CD8^+^ T cells was higher in peri-tumor cores than in tumor cores (*P* < 0.0001). In addition, the density of CD68^+^ macrophages was more abundant in the tumor than in the peri-tumor (*P*<0.0001) (Fig. 1b).

The association between the clinicopathological factors and CD8^+^ T cells/CD68^+^ macrophages was presented in Table 1. Youden index was used to categorize high and low expression of CD8 and CD68. High density of CD8^+^ T cells correlated with lower CA19-9 (*P* = 0.014), lower Child-Pugh stage (*P* = 0.027), and smaller tumor size (*P* = 0.009). High density of CD68^+^ macrophages correlated with smaller tumor size (*P* = 0.003) and tumor number (*P* = 0.014) (Table 1). Furthermore, patients with high density of CD8^+^ T cells has better OS (logrank test: *P* = 0.006) and favorable TTR (logrank test: *P* = 0.003). But CD68^+^ macrophages failed to stratify the OS (logrank test: *P* = 0.1095) and TTR (logrank test: *P* = 0.6781) (Fig. 1c, d). In the multivariate analyses, CD8^+^ T cells were an independent prognostic factor of OS (*P* = 0.002, HR = 0.629, 95% CI 0.470–0.842) and TTR (*P* = 0.001, HR = 0.614, 95% CI 0.454–0.848) (Table 2, Supplementary Table 2).

To sum up, these data identified that CD8^+^ T cells were valuable independent risk factors for both OS and TTR.

### The characterization and prognostic implication of PD-1 expression on CD8^+^ T cells

**Fig. 2:**
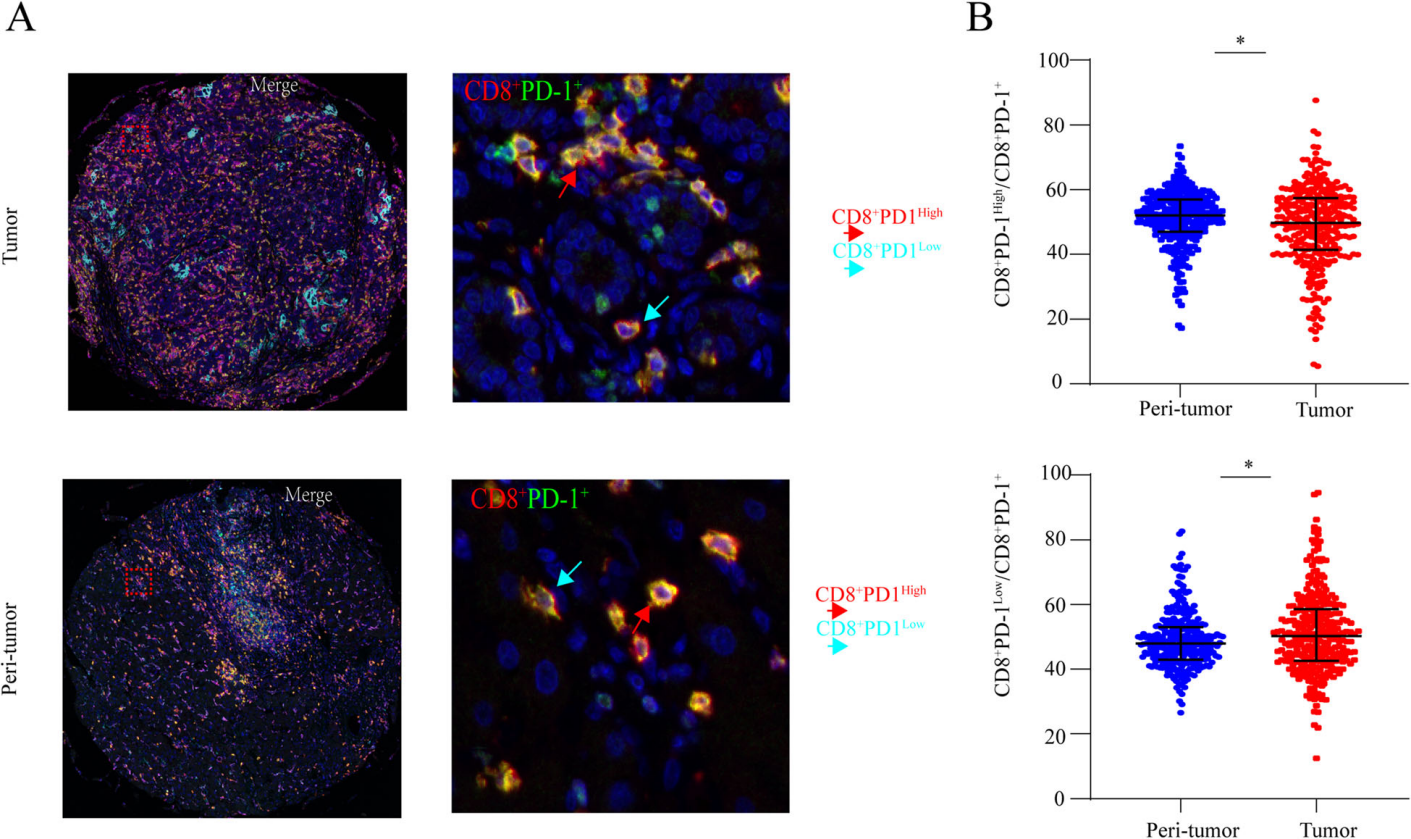
Different expression patterns of PD-1 in CTLs in ICC tumors. **a** Representative multiplex immunofluorescence staining to reveal different expression level of PD1 on both CD8 in tumor tissues (T) and peri-tumor tissues (P): red arrows (CD8^+^ PD-1^High^); yellow arrows (CD8^+^ PD-1^Low^). Scale bar, 50 μm. High expression and low expression in CTLs in tumor tissue were observed by double color staining. **b** Quantitative comparisons analysis of CD8^+^ PD-1^High^ cells and CD8^+^ PD-1^Low^ cells in between the paired tumor tissues (T) and peri-tumor tissues (P) (Wilcoxon signed-rank test)

**Fig. 3:**
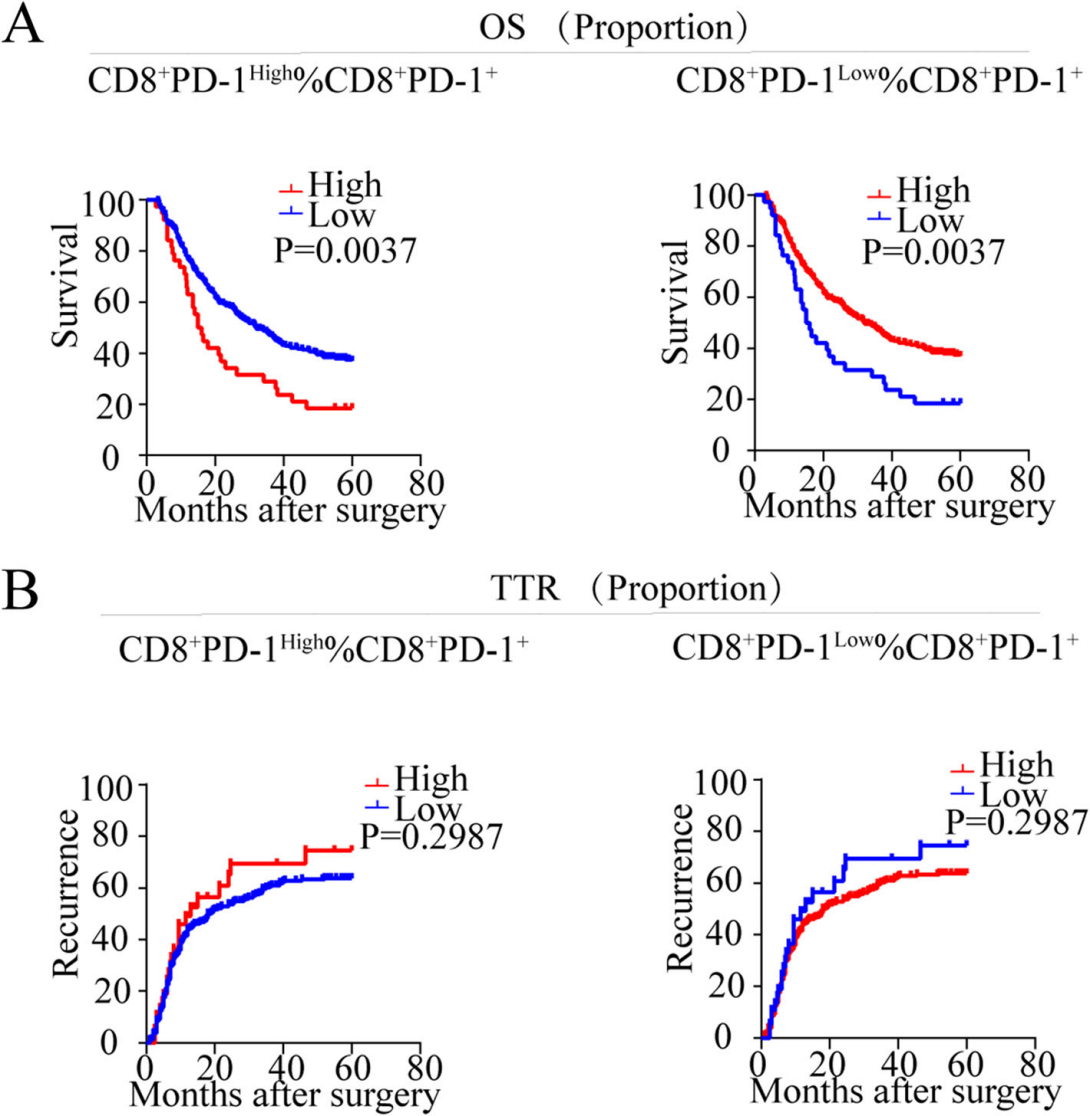
Prognostic implication of different expression patterns of PD-1 in CD8^+^ T cells in ICC patients. **a**, **b** Kaplan-Meier analysis of overall survival (OS) (**a**) and relapse-free survival (RFS) (**b**) in ICC according to the frequency of CD8^+^ PD-1^High^ within CD8^+^ PD-1^+^, CD8^+^ PD-1^Low^ within CD8^+^ PD-1^+^

As illustrated in Supplementary Figure 1B, the PD-1 expression is positively correlated with the infiltration of CD8^+^ T cells in both tumor (*P* < 0.0001, *r* = 0.5790) and peri-tumor (*P* < 0.0001, *r* = 0.9375). Subsequently, we analyzed the staining of PD-1 and CD8 in tumor and peri-tumor cores which could divide the sample into two subgroups by PD-1 expression on CD8^+^ T cells. Therefore, the cut-off value was applied to classify the patients into CD8^+^ PD-1^High^ and CD8^+^ PD-1^Low^ according to the expression level of PD-1 (Fig. 2a). The proportion of CD8^+^ PD-1^High^ cells within CD8^+^ PD-1^+^ T cells was higher in peri-tumor cores than in tumor cores (*P* = 0.011), and the proportion of CD8^+^ PD-1^Low^ cells within the CD8^+^ PD-1^+^ T cells was higher in tumor cores than in peri-tumor cores (*P* = 0.011) (Fig. 2b). Furthermore, the proportion of CD8^+^ PD-1^High^ (*P* = 0.035) within CD8^+^ PD-1^+^ T cells in tumor correlated with advanced TNM stage (Supplementary Table 3). ICC patients with high proportion of CD8^+^ PD-1^High^ in tumor had worse OS than low proportion patients (logrank test: *P* = 0.0037). In addition, the proportion of CD8^+^ PD-1^High^ (logrank test: *P* = 0.035) in tumor failed to stratify the TTR in ICC patients (Fig. 3). Univariate and multivariate analyses showed the proportion of CD8^+^ PD-1^High^ (*P* = 0.025, HR = 1.557, 95% CI 1.057–2.292) within CD8^+^ PD-1^+^ T cells in tumor was the independent prognostic factor for OS in ICC patients (Table 2).

From the data above, we can conclude that CD8^+^ PD-1^High^ within CD8^+^ PD-1^+^ T cells in tumor was independent prognostic factor for postoperative survival.

### The prevalence CD68^+^ PD-L1^+^ macrophages cells and their correlation with CD8^+^ PD-1^High^ T cells

**Fig. 4:**
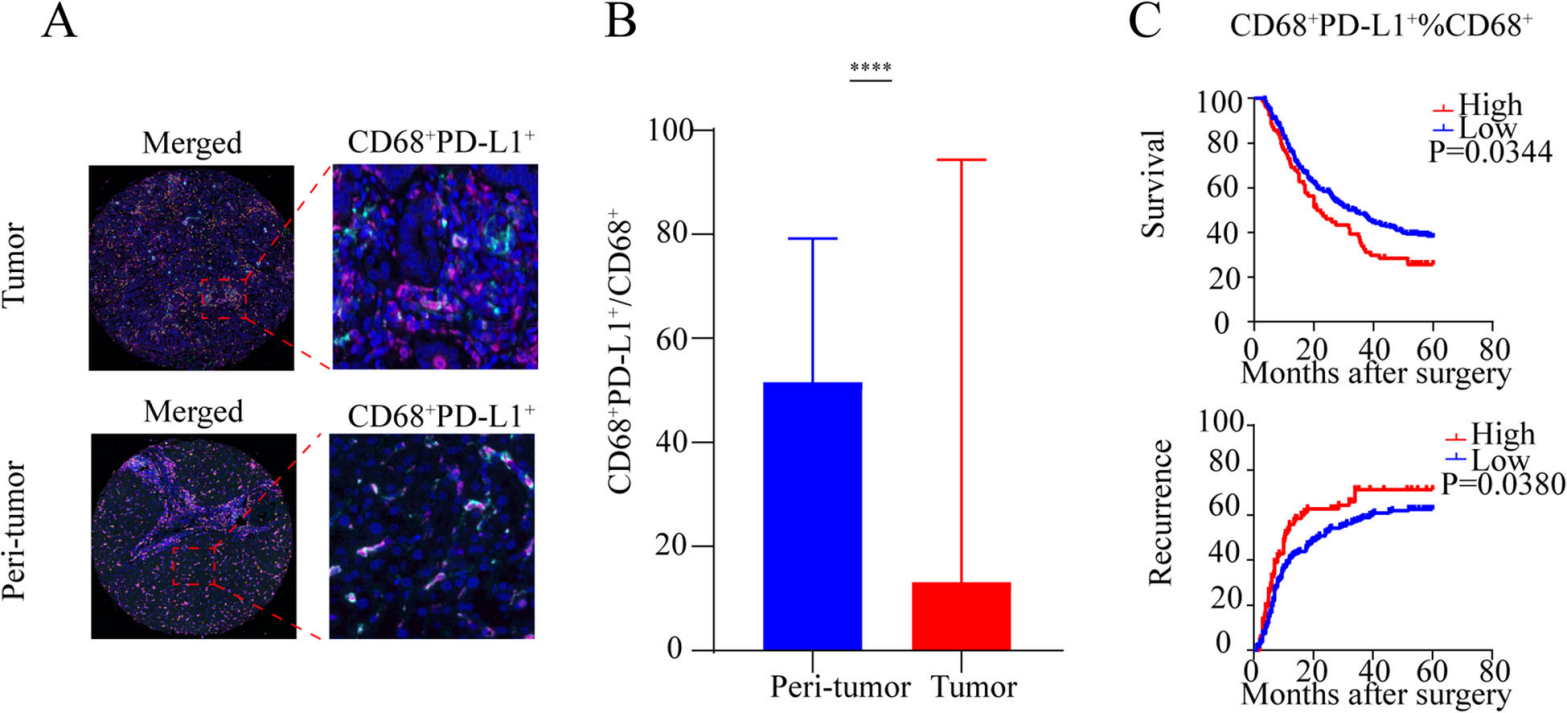
Different expression patterns of PD-L1 in CD68^+^ macrophages in ICC tumors and prognostic implication in ICC patients. **a** Representative multiplex immunofluorescence staining with the indicated immune markers of CD68^+^ PD-L1^+^ in ICC tumor and peri-tumor tissues. Scale bar, 50 μm. **b** Quantitative comparisons analysis of the proportion of CD68^+^ PD-L1^+^ between paired tumor tissues (T) and peri-tumor tissues (P) in the ICC patients (Wilcoxon signed-rank test). **c** Kaplan-Meier analysis of overall survival (OS) and relapse-free survival (RFS) in ICC according to the frequency of CD68^+^ PD-L1 within CD68^+^ PD-1^+^

The presence of CD68^+^ PD-L1^+^ macrophages was illustrated in Fig. 4a for both tumor tissue and peri-tumor tissue. The proportion of CD68^+^ PD-L1^+^ cells within CD68^+^ macrophages was higher in peri-tumor (*P* < 0.0001) (Fig. 4b).The proportion of CD68^+^ PD-L1^+^ within CD68^+^ macrophages correlated with aggressive the clinicopathological factors such as high level of CA19-9 (*P* = 0.027), higher Child-Pugh stage (*P* = 0.017), lymph node metastasis (*P* = 0.027), microscopic vascular invasion (*P* = 0.029), and advanced TNM stage (*P* = 0.036) (Supplementary Table 3). High proportion of CD68^+^ PD-L1^+^ cells within CD68^+^ macrophages in ICC patients had shorter OS (logrank test: *P* = 0.0344) and worse TTR (logrank test: *P* = 0.0380) (Fig. 4c). In the multivariate analyses, the proportion of CD68^+^ PD-L1^+^ cells within CD68^+^ macrophages in ICC failed to be an independent prognostic factor for OS and TTR in ICC patients (Table 2, Supplementary Table 2).

Furthermore, we found that the density of CD68^+^ PD-L1^+^ macrophages positively correlated with the density of CD8^+^ PD-1^High^ T cells (*P* < 0.0001, *r* = 0.5927) (Supplementary Figure 1C). As a whole, the CD68^+^ PD-L1^+^ macrophages and CD8^+^ PD-1^High^ T cells may cooperatively play a role in inhibiting anti-tumor immunity.

## Discussion

Currently, immune checkpoints within the TME have been identified as potential therapeutic targets, but the role of PD-1/PD-L1 in the TME remains largely undetermined for ICC [26]. This investigation is to simultaneously analyze the expression patterns of PD-1/PD-L1 in CD8^+^ T cells and CD68^+^ macrophages, as well as examine their association with the clinical significance of ICC patients. Our analyses provide further investigation into the significance of the TME in the prognoses of solid tumors.

Our study found that the expression of PD-1 in ICC tissues correlated with better OS. The favorable value of PD-1 has also been reported in other solid tumors, such as ovarian cancer [27]. Furthermore, high expression of PD-1 was positively correlated with lower CA19-9 level, smaller tumor size, and HBsAg in our research, and it has been shown that CA19-9 could predict postoperative survival for ICC patients in a previous study [28]. In our study, it is found that PD-L1 expression was higher in tumor tissues than in peri-tumor tissues (*P* = 0.0123), which is consistent with previous studies [29]. Our data illustrated that the expression of PD-L1 in ICC tumors is not an independent prognostic factor, but the different prognosis of PD-L1 expression is reported in other studies. A research by Tan et al. suggests that high PD-L1 expression is an indicator of a favorable prognosis in ovarian cancer [27]. However, Jung et al. reported the opposite result, whereby over-expression of PD-L1 in liver cancer suggests a poor prognosis [30].

To explore PD-1 expression in CD8^+^ T cells in the TME, multicolor-staining was utilized to assess the expression of PD-1 in immune cells, using PD-1 and CD8 by mIHC to analyze simultaneously, which helps to make predictions and accurately stratify patients compared with only CD8 staining or only PD-1 staining in ICC study [31]. We further demonstrated that the percentage of CD8^+^ PD-1^High^ and CD8^+^ PD-1^Low^ in CD8^+^ PD-1^+^ are independent prognostic factors for OS in patients with ICC. Patients with a high percentage of CD8^+^ PD-1^High^ had a poor postoperative survival. This is consistent with previous reports from other tumors, such as gastric cancer [32], pancreatic cancer [33], and breast cancer [34]. This may be due to PD-1^High^ expression indicating CD8^+^ T cells are highly activated, but exhibit a severe dysfunction phenotype and impaired IFN-γ secretion and negatively affect clinical outcomes [35]. Our results demonstrate that among the activated CD8^+^ PD-1^+^ cells, a high proportion of CD8^+^ PD-1^High^ causes exhaustion of CD8^+^ T cells and a poor prognosis. CD8^+^ PD-1^High^ T cells comprised TNF-α and IL-2 expression, indicating that the production of cytokines and anti-tumor ability were generally defective, while CD8^+^ PD-1^High^ T cells upregulated the expression of immunosuppressive cytokine IL-10, suggesting that CD8^+^ PD-1^High^ T cells may obtain the ability to inhibit the immune response in HCC [17]. CD8^+^ PD-1^High^ T cells in human lung cancer shows profound metabolic changes, and the limitation of metabolic intermediates necessary for antitumor activity may further increase the dysfunction of CD8^+^ PD-1^High^ T cells [36]. Moreover, when the proportion of CD8^+^ PD-1^Low^ in CD8^+^ PD-1^+^ is low, patients with less CD8^+^ T cell exhaustion have a better prognosis. Targeting the PD-1 may block the suppression of anti-cancer immunity by inhibiting the exhaustion of CD8^+^ T cells and reverse the dysfunction of tumor-infiltrating lymphocytes in HCC [37]. In ICC, it is also suggested that blocking PD-1 could be a potential target [38].

The expression of CD68 or PD-L1 has no prognostic value in our study, respectively, which is consistent with the previous result [10, 13]. Some reports suggest that PD-L1 is expressed in tumor cells and immune cells, and it has different biological and clinical significance, which may be the loss of prognostic value due to the different expression of PD-L1 in different cells [39]. We further explore the prognostic significance of PD-L1 expression on macrophages, rather than the overall analysis of PD-L1 expression and macrophage infiltration as. In our study, the expression of PD-L1 on CD68^+^ macrophages has a frustrated role for both postoperative survival and recurrence for ICC patients. One study in oral squamous cell carcinoma showed that PD-L1 expression on TAMs suppressed the anti-tumor immunity, which is in line with our study [40]. Intriguingly, Zheng et al. found that PD-L1^+^ expressed on TAMs correlated with better prognosis of HCC patients because TAMs-PD-L1^+^ tumors showed a high expression of genes involved in the immune responses and lymphocyte activation leading to anti-tumor activity in the TME [39]. The results of the contradiction may be due to the opposite effects of M1 and M2 macrophages [41].

More interestingly, we found that the expression of CD68^+^ PD-L1^+^ was correlated with the expression of CD8^+^ PD-1^High^, and both CD68^+^ PD-L1^+^ cells and CD8^+^ PD-1^High^ cells had unfavorable prognosis. One similar study in HCC found that there is an intimate spatial relationship between the CD8^+^ exhausted T cells and PD-L1^+^ tumor-associated macrophages in HCC TME [17]. Thus, we speculate that the interaction of the CD68^+^ PD-L1^+^ cells and CD8^+^ PD-1^High^ cells plays a role in inhibiting the anti-cancer response. PD-L1 engenders constitutive signaling impacts on macrophages, resulting in restraint of activation and decreasing survival, and PD-L1 antibodies can reshape the macrophages to enhance their ability to secrete the inflammatory cytokine and promote T cell proliferation and activation [42–44].

The current study has several limitations. First, the data in this study was derived from only one hepatobiliary center. Second, only CD8^+^ T cells and CD68^+^ macrophages were taken into consideration in our study, which does not reflect all the characteristics of the immune environment of ICC; other kinds of immune cells should be taken into consideration in the future. Finally, a prospective study is required to validate the results of this retrospective study. In future research, we will expand the varieties of immune cells and conduct research across multiple hepatobiliary centers.

In summary, a high percentage of CD8^+^ PD-1^High^ serves as an unfavorable prognostic factor in patients with ICC. High expression of CD8^+^ PD-1^High^ is associated with CD68^+^ PD-L1^+^, suggesting that CD68^+^ PD-L1^+^ could cooperate with CD8^+^ PD-1^High^ to cause the suppression of anti-cancer immunity.

## Supplementary Information

**Additional file 1:** **Supplementary Figure 1**. **A** CD8 and CD68 cell subsets are defined by four-color multiplexed immunohistochemistry in HCC. Digital scanning displayed bright-field image and multispectral image (MSI) of one TMA core from ICC tumor or peri-tumor tissues. **B** Correlation of the density of CD8^+^ T cells and PD-1^+^ in the tumor. **C** Correlation of the density of CD68^+^ PD-L1^+^ and CD8^+^ PD-1^High^ in the tumor

**Additional file 2:** **Supplementary Table 1**. Patient characteristics in 322 patients

**Additional file 3:** **Supplementary Table 2**. Univariate and multivariate analyses of prognostic features and time to recurrence

**Additional file 4:** **Supplementary Table 3**. Correlation between the proportion of CD8^+^ PD-1^High^, CD8^+^ PD-1^Low^, CD68^+^ PD-L1 and clinicopathological features

## Data Availability

Please contact the corresponding authors if necessary.

## References

[1] Dodson RM (2013). Intrahepatic cholangiocarcinoma: management options and emerging therapies. J Am Coll Surg.

[2] Razumilava N, Gores GJ (2014). Cholangiocarcinoma. Lancet.

[3] Welzel TM (2007). Risk factors for intrahepatic and extrahepatic cholangiocarcinoma in the United States: a population-based case-control study. Clin Gastroenterol Hepatol.

[4] Zhou YM (2008). Risk factors for intrahepatic cholangiocarcinoma: a case-control study in China. World J Gastroenterol.

[5] Banales JM (2016). Expert consensus document: Cholangiocarcinoma: current knowledge and future perspectives consensus statement from the European Network for the Study of Cholangiocarcinoma (ENS-CCA). Nat Rev Gastroenterol Hepatol.

[6] Weber SM (2015). Intrahepatic cholangiocarcinoma: expert consensus statement. HPB (Oxford).

[7] Vesely MD (2011). Natural innate and adaptive immunity to cancer. Annu Rev Immunol.

[8] Hanahan D, Weinberg RA (2011). Hallmarks of cancer: the next generation. Cell.

[9] Smyth MJ (2016). Combination cancer immunotherapies tailored to the tumour microenvironment. Nat Rev Clin Oncol.

[10] Jing CY (2019). HHLA2 in intrahepatic cholangiocarcinoma: an immune checkpoint with prognostic significance and wider expression compared with PD-L1. J Immunother Cancer.

[11] Gajewski TF, Schreiber H, Fu YX (2013). Innate and adaptive immune cells in the tumor microenvironment. Nat Immunol.

[12] Fabris L (2019). The tumour microenvironment and immune milieu of cholangiocarcinoma. Liver Int.

[13] Vigano L (2019). Tumor-infiltrating lymphocytes and macrophages in intrahepatic cholangiocellular carcinoma. Impact on prognosis after complete surgery. J Gastrointest Surg.

[14] Atanasov G (2017). Tumor necrosis and infiltrating macrophages predict survival after curative resection for cholangiocarcinoma. Oncoimmunology.

[15] Oishi K (2015). Macrophage density and macrophage colony-stimulating factor expression predict the postoperative prognosis in patients with intrahepatic cholangiocarcinoma. Surg Today.

[16] Santabarbara G (2016). Novel immunotherapy in the treatment of advanced non-small cell lung cancer. Expert Rev Clin Pharmacol.

[17] Ma J (2019). PD1(Hi) CD8(+) T cells correlate with exhausted signature and poor clinical outcome in hepatocellular carcinoma. J Immunother Cancer.

[18] Gordon SR (2017). PD-1 expression by tumour-associated macrophages inhibits phagocytosis and tumour immunity. Nature.

[19] Gani F (2016). Program death 1 immune checkpoint and tumor microenvironment: implications for patients with intrahepatic cholangiocarcinoma. Ann Surg Oncol.

[20] Sui M (2019). Two cases of intrahepatic cholangiocellular carcinoma with high insertion-deletion ratios that achieved a complete response following chemotherapy combined with PD-1 blockade. J Immunother Cancer.

[21] Liu LZ (2016). Protein tyrosine phosphatase PTP4A1 promotes proliferation and epithelial-mesenchymal transition in intrahepatic cholangiocarcinoma via the PI3K/AKT pathway. Oncotarget.

[22] Liu LZ (2018). CK7/CK19 index: a potential prognostic factor for postoperative intrahepatic cholangiocarcinoma patients. J Surg Oncol.

[23] Gao Q (2009). Overexpression of PD-L1 significantly associates with tumor aggressiveness and postoperative recurrence in human hepatocellular carcinoma. Clin Cancer Res.

[24] Yang LX (2015). Mitogen-activated protein kinase kinase kinase 4 deficiency in intrahepatic cholangiocarcinoma leads to invasive growth and epithelial-mesenchymal transition. Hepatology.

[25] Gorris MAJ (2018). Eight-color multiplex immunohistochemistry for simultaneous detection of multiple immune checkpoint molecules within the tumor microenvironment. J Immunol.

[26] Soares KC (2015). PD-1/PD-L1 blockade together with vaccine therapy facilitates effector T-cell infiltration into pancreatic tumors. J Immunother.

[27] Tan D, Sheng L, Yi QH (2018). Correlation of PD-1/PD-L1 polymorphisms and expressions with clinicopathologic features and prognosis of ovarian cancer. Cancer Biomark.

[28] Zheng BH (2017). A new preoperative prognostic system combining CRP and CA199 for patients with intrahepatic cholangiocarcinoma. Clin Transl Gastroenterol.

[29] Ma K (2017). PD-L1 and PD-1 expression correlate with prognosis in extrahepatic cholangiocarcinoma. Oncol Lett.

[30] Jung HI (2017). Overexpression of PD-L1 and PD-L2 is associated with poor prognosis in patients with hepatocellular carcinoma. Cancer Res Treat.

[31] Ying L (2017). Understanding immune phenotypes in human gastric disease tissues by multiplexed immunohistochemistry. J Transl Med.

[32] Saito H (2018). Highly activated PD-1/PD-L1 pathway in gastric cancer with PD-L1 expression. Anticancer Res.

[33] Shen T (2017). Prognostic value of programmed cell death protein 1 expression on CD8+ T lymphocytes in pancreatic cancer. Sci Rep.

[34] Muenst S (2013). The presence of programmed death 1 (PD-1)-positive tumor-infiltrating lymphocytes is associated with poor prognosis in human breast cancer. Breast Cancer Res Treat.

[35] Kansy BA (2017). PD-1 Status in CD8(+) T cells associates with survival and anti-PD-1 therapeutic outcomes in head and neck cancer. Cancer Res.

[36] Thommen DS (2018). A transcriptionally and functionally distinct PD-1(+) CD8(+) T cell pool with predictive potential in non-small-cell lung cancer treated with PD-1 blockade. Nat Med.

[37] Liu F (2018). Blocking Tim-3 or/and PD-1 reverses dysfunction of tumor-infiltrating lymphocytes in HBV-related hepatocellular carcinoma. Bull Cancer.

[38] Liu X (2019). Local and abscopal responses in advanced intrahepatic cholangiocarcinoma with low TMB, MSS, pMMR and negative PD-L1 expression following combined therapy of SBRT with PD-1 blockade. J Immunother Cancer.

[39] Liu CQ (2018). Expression patterns of programmed death ligand 1 correlate with different microenvironments and patient prognosis in hepatocellular carcinoma. Br J Cancer.

[40] Jiang C (2017). Oral squamous cell carcinoma suppressed antitumor immunity through induction of PD-L1 expression on tumor-associated macrophages. Immunobiology.

[41] Cao L (2019). M2 macrophage infiltration into tumor islets leads to poor prognosis in non-small-cell lung cancer. Cancer Manag Res.

[42] Hartley GP (2018). Programmed cell death ligand 1 (PD-L1) signaling regulates macrophage proliferation and activation. Cancer Immunol Res.

[43] Pardoll DM (2012). The blockade of immune checkpoints in cancer immunotherapy. Nat Rev Cancer.

[44] Xiong H (2019). Anti-PD-L1 treatment results in functional remodeling of the macrophage compartment. Cancer Res.

