# Supplementary figures and images for: PD-1/PD-L1 expression profiles within intrahepatic cholangiocarcinoma predict clinical outcome
Source: World J Surg Oncol. 2020 Nov 23;18:303. doi: 10.1186/s12957-020-02082-5 (PMC7686719; doi:10.1186/s12957-020-02082-5)

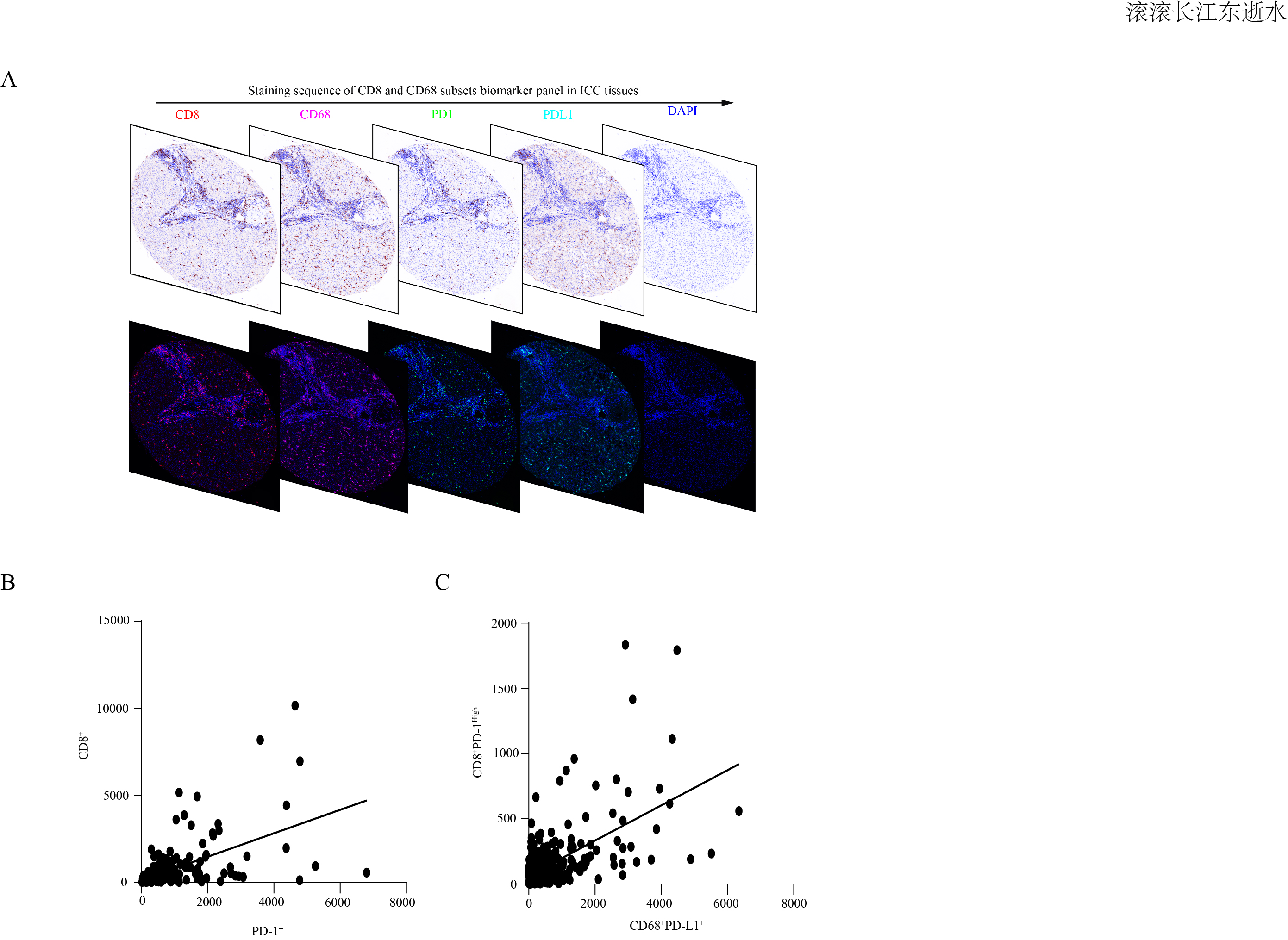

Supplement: Supplementary file 1 — Additional file 1: Supplementary Figure 1. A CD8 and CD68 cell subsets are defined by four-color multiplexed immunohistochemistry in HCC. Digital scanning displayed bright-field image and multispectral image (MSI) of one TMA core from ICC tumor or peri-tumor tissues. B Correlation of the density of CD8+ T cells and PD-1+ in the tumor. C Correlation of the density of CD68+ PD-L1+ and CD8+ PD-1High in the tumor [file 12957_2020_2082_MOESM1_ESM.jpg]
