# Supplementary material for: PD-1/PD-L1 expression profiles within intrahepatic cholangiocarcinoma predict clinical outcome
Source: World J Surg Oncol. 2020 Nov 23;18:303. doi: 10.1186/s12957-020-02082-5 (PMC7686719; doi:10.1186/s12957-020-02082-5)
Supplement: Supplementary file 2 — Additional file 2: Supplementary Table 1. Patient characteristics in 322 patients [file 12957_2020_2082_MOESM2_ESM.docx]

**Supplementary Table 1. Patient characteristics in 322 patients.**

| **Variable** | **Results** |
| --- | --- |
| **Age (years)** |  |
| ＜60 vs.≥60 | 173/149 |
| **Gender** |  |
| Male vs.Female | 194/128 |
| **HBsAg** |  |
| Negative vs. Positive | 199/123 |
| **Cirrhosis** |  |
| No vs. Yes | 236/86 |
| **CA-199** |  |
| Low (≤37) vs.High (＞37) | 163/159 |
| **Child-Pugh** |  |
| A vs.B | 311/11 |
| **Size(cm)** |  |
| ≤5 vs. >5 | 145/177 |
| **Number** |  |
| Single vs. Multiple | 244/78 |
| **LN invasion** |  |
| No vs. Yes | 266/56 |
| **TNM stage** |  |
| I vs. II-III | 247/75 |
| **MVI** |  |
| No vs. Yes | 276/46 |

Abbreviation: *HBsAg,* Hepatitis B Surface antigen; *CA19-9,* Carbohydrate antigen 19-9; *LN,* Lymph node; *TNM,* Tumor-Nodes-Metastasis; *MVI,* Microvascular invasion
