# Supplementary material for: PD-1/PD-L1 expression profiles within intrahepatic cholangiocarcinoma predict clinical outcome
Source: World J Surg Oncol. 2020 Nov 23;18:303. doi: 10.1186/s12957-020-02082-5 (PMC7686719; doi:10.1186/s12957-020-02082-5)
Supplement: Supplementary file 3 — Additional file 3: Supplementary Table 2. Univariate and multivariate analyses of prognostic features and time to recurrence [file 12957_2020_2082_MOESM3_ESM.docx]

**Supplementary Table 2. Univariate and multivariate analyses of prognostic features and time to recurrence.**

|  | **TTR** | | | |
| --- | --- | --- | --- | --- |
| **Variable** | **Univariate analysis** | | **Multivariate analyses** | |
|  | HR (95%CI) | P | HR (95%CI) | P |
| **Age (years)** |  |  |  |  |
| ＜60 vs.≥60 | 0.870(0.653-1.159) | 0.342 |  |  |
| **Gender** |  |  |  |  |
| Male vs.Female | 1.130(0.843-1.515) | 0.412 |  |  |
| **HBsAg** |  |  |  |  |
| Negative vs. Positive | 0.867(0.646-1.164) | 0.343 |  |  |
| **Cirrhosis** |  |  |  |  |
| No vs. Yes | 1.211(0.882-1.663) | 0.237 |  |  |
| **CA19-9** |  |  |  |  |
| Low (≤37) vs.High (＞37) | 1.285(0.967-1.709) | 0.084 |  |  |
| **Child-Pugh** |  |  |  |  |
| A vs.B | 0.535(0.199-1.441) | 0.216 |  |  |
| **Size(cm)** |  |  |  |  |
| ≤5 vs. >5 | 1.504(1.125-2.009) | **0.006** |  |  |
| **Number** |  |  |  |  |
| Single vs. Multiple | 1.783(1.302-2.442) | **0.000** |  |  |
| **LN invasion** |  |  |  |  |
| No vs. Yes | 2.293(1.621-3.245) | **0.000** |  |  |
| **TNM stage** |  |  |  |  |
| I vs. II-III | 2.021(1.473-2.772) | **0.000** |  |  |
| **MVI** |  |  |  |  |
| No vs. Yes | 1.525(1.052-2.210) | **0.026** |  |  |
|  |  |  |  |  |
| **PD-1** |  |  |  |  |
| Low vs. High | 0.504(0.367-0.691) | **0.000** | 0.554(0.402-0.763) | **0.000^a^** |
| **PD-L1** |  |  |  |  |
| Low vs. High | 1.285(0.936-1.764) | 0.121 |  |  |
| **CD8** |  |  |  |  |
| Low vs. High | 0.587(0.436-0.790) | **0.000** | 0.614(0.454-0.828) | **0.001^b^** |
| **CD68** |  |  |  |  |
| Low vs. High | 0.945(0.709-1.261) | 0.702 |  |  |
| **CD8PD1^High^/CD8PD1** |  |  |  |  |
| Low vs. High | 1.241(0.802-1.921) | 0.333 |  |  |
| **CD8PD1^Low^/CD8PD1** |  |  |  |  |
| Low vs. High | 0.806(0.521-1.247) | 0.333 |  |  |
| **CD68PDL1/CD68** |  |  |  |  |
| Low vs. High | 1.388(1.011-1.906) | **0.043** | 1.217(0.880-1.682) | 0.235^c^ |

Abbreviation: *HBsAg,* Hepatitis B Surface antigen; *CA19-9,* Carbohydrate antigen 19-9; *LN,* Lymph node; *TNM,* Tumor-Nodes-Metastasis; *MVI,* Microvascular invasion

Cox regression model was performed;

*a,b,and c. Size，Number，LN invasion，TNM stage and MVI* were adjusted in multivariate analyse.
