# Supplementary material for: PD-1/PD-L1 expression profiles within intrahepatic cholangiocarcinoma predict clinical outcome
Source: World J Surg Oncol. 2020 Nov 23;18:303. doi: 10.1186/s12957-020-02082-5 (PMC7686719; doi:10.1186/s12957-020-02082-5)
Supplement: Supplementary file 4 — Additional file 4: Supplementary Table 3. Correlation between the proportion of CD8+ PD-1High, CD8+ PD-1Low, CD68+ PD-L1 and clinicopathological features [file 12957_2020_2082_MOESM4_ESM.docx]

**Supplementary Table 3. Correlation between the proportion of CD8^+^PD-1^High^，CD8^+^PD-1^Low^，**

**CD68^+^PD-L1 and clinicopathological features.**

| Characteristics | CD8^+^PD-1^High^% | | | CD8^+^PD-1^Low^% | | | CD68^+^PD-L1^+^% | | |
| --- | --- | --- | --- | --- | --- | --- | --- | --- | --- |
|  | Low | High | P | Low | High | P | Low | High | P |
| Age, years |  |  |  |  |  |  |  |  |  |
| ＜60 | 152 | 21 | 0.840 | 21 | 152 | 0.840 | 130 | 43 | 0.776 |
| ≥60 | 132 | 17 |  | 17 | 132 |  | 114 | 35 |  |
| Gender |  |  |  |  |  |  |  |  |  |
| Female | 114 | 14 | 0.696 | 14 | 114 | 0.696 | 108 | 20 | **0.003** |
| Male | 170 | 24 |  | 24 | 170 |  | 136 | 58 |  |
| HBsAg |  |  |  |  |  |  |  |  |  |
| Negative | 173 | 26 | 0.371 | 26 | 173 | 0.371 | 150 | 49 | 0.831 |
| Positive | 111 | 12 |  | 12 | 111 |  | 94 | 29 |  |
| Cirrhosis |  |  |  |  |  |  |  |  |  |
| No | 208 | 28 | 0.954 | 28 | 208 | 0.954 | 182 | 54 | 0.352 |
| Yes | 76 | 10 |  | 10 | 76 |  | 62 | 24 |  |
| CA19-9 |  |  |  |  |  |  |  |  |  |
| Low (≤37) | 148 | 15 | 0.143 | 15 | 148 | 0.143 | 132 | 31 | **0.027** |
| High (>37) | 136 | 23 |  | 23 | 136 |  | 112 | 47 |  |
| Child-pugh |  |  |  |  |  |  |  |  |  |
| A | 274 | 37 | 0.777 | 37 | 274 | 0.777 | 239 | 72 | **0.017** |
| B | 10 | 1 |  | 1 | 10 |  | 5 | 6 |  |
| Tumor size (cm) |  |  |  |  |  |  |  |  |  |
| ≤5 | 129 | 16 | 0.699 | 16 | 129 | 0.699 | 115 | 30 | 0.180 |
| >5 | 155 | 22 |  | 22 | 155 |  | 129 | 48 |  |
| Tumor number |  |  |  |  |  |  |  |  |  |
| Single | 217 | 27 | 0.469 | 27 | 217 | 0.469 | 189 | 55 | 0.213 |
| Multiple | 67 | 11 |  | 11 | 67 |  | 55 | 23 |  |
| LN invasion |  |  |  |  |  |  |  |  |  |
| No | 237 | 29 | 0.276 | 29 | 237 | 0.276 | 208 | 58 | **0.027** |
| Yes | 47 | 9 |  | 9 | 47 |  | 36 | 20 |  |
| TNM stage |  |  |  |  |  |  |  |  |  |
| I | 223 | 24 | **0.035** | 24 | 223 | **0.035** | 194 | 53 | **0.036** |
| II-III | 61 | 14 |  | 14 | 61 |  | 50 | 25 |  |
| MVI |  |  |  |  |  |  |  |  |  |
| No | 244 | 32 | 0.778 | 32 | 244 | 0.778 | 215 | 61 | **0.029** |
| Yes | 40 | 6 |  | 6 | 40 |  | 29 | 17 |  |

Abbreviation: *HBsAg,* Hepatitis B Surface antigen; *CA19-9,* Carbohydrate antigen 19-9;

*LN,* Lymph node; *TNM,* Tumor-Nodes-Metastasis; *MVI,* Microvascular invasion

Chi-square test, Fisher exact test was performed;
